# Targeting iron metabolism in osteosarcoma

**DOI:** 10.1007/s12672-023-00637-y

**Published:** 2023-03-10

**Authors:** Xiaowei Ma, Jiazheng Zhao, Helin Feng

**Affiliations:** 1 Department of Orthopedics, National Cancer Center/National Clinical Research Center for Cancer/Cancer Hospital, Chinese Academy of Medical Sciences and Peking Union Medical College, No. 17 Nanli, Panjiayuan, Chaoyang District, Beijing, 100021 People’s Republic of China; 2 Department of Orthopedics, The Fourth Hospital of Hebei Medical University, 12 Health Road, Hebei Province Shijiazhuang, 050011 People’s Republic of China

**Keywords:** Osteosarcoma, Reactive oxygen species, Iron metabolism, TfR1

## Abstract

Osteosarcoma (OS) is the most common primary solid malignant tumour of bone, with rapid progression and a very poor prognosis. Iron is an essential nutrient that makes it an important player in cellular activities due to its inherent ability to exchange electrons, and its metabolic abnormalities are associated with a variety of diseases. The body tightly regulates iron content at the systemic and cellular levels through various mechanisms to prevent iron deficiency and overload from damaging the body. OS cells regulate various mechanisms to increase the intracellular iron concentration to accelerate proliferation, and some studies have revealed the hidden link between iron metabolism and the occurrence and development of OS. This article briefly describes the process of normal iron metabolism, and focuses on the research progress of abnormal iron metabolism in OS from the systemic and cellular levels.

## Introduction

OS is the most common primary solid malignant tumor of bone. It is a highly malignant tumor derived from mesenchymal tissue with unique clinical and pathological characteristics. The incidence of OS in the general population is 2–3/million/year, but the incidence is higher in adolescents. At the age of 15–19, the annual incidence rate is as high as 8–11/million/year [1], and it is the second leading cause of cancer-related deaths in children and young people [2]. The occurrence of OS is a complex process involving multiple factors; it is characterized by rapid disease progression, high malignancy, and easy recurrence and metastasis. Even after the standard treatment regimen of OS, the survival rate of OS without distant metastasis is 70%, while the 5-year survival rate of metastatic OS is only 20% to 30% [3].

As a vital nutrient element in human life activities, iron is an important participant in cell proliferation and growth, including mitochondrial function, and an essential cofactor in oxidative phosphorylation in the aerobic respiration chain of cells. Iron is involved in the synthesis of hemoglobin and DNA synthesis and repair and other important life activities [4, 5]. Therefore, iron is essential for cell growth and proliferation. In addition, iron can gain and lose electrons, and iron overload promotes the production of reactive oxygen species (ROS). ROS not only damage proteins and lipids but can also damage DNA and cause DNA mutations to promote tumorigenesis [6–8]. A large number of studies suggest that intracellular iron metabolism disorders are related to the occurrence and development of tumors [9–12]. In this review, we first briefly introduce the characteristics of normal iron metabolism in human body, and also expounds the research progress of abnormal iron metabolism in OS from the systemic and cellular levels.

## Normal iron metabolism

Iron metabolism in the body includes the absorption, storage, transport, utilization and excretion of iron. The body iron content of a normal healthy adult is between 3–4 g, and 60–70% of iron is present in the haemoglobin of red blood cells [13]. Approximately 10% of the iron required for normal physiological activities is absorbed from food by enterocytes and 90% comes from the reuse of red blood cells. An appropriate amount of iron is essential for the survival, growth and reproduction of cells, while excessive iron potentially damages cells. Therefore, the process of iron uptake, storage and excretion has a strict regulatory mechanism.

### Iron absorption and import

In the intestine, iron (Fe^3+^) in its oxidized state in food is reduced to Fe^2+^ by iron reductase (duodenal cytochrome B, DcytB) and then taken up by the divalent metal transporter 1 (DMT1) into intestinal epithelial cells [14, 15]. Heme carrier protein 1 (HCP1) is highly expressed on the brush-like margin of intestinal epithelial cells in the duodenum, and HCP1 mediates the absorption of heme iron by intestinal cells [16].

**Fig. 1:**
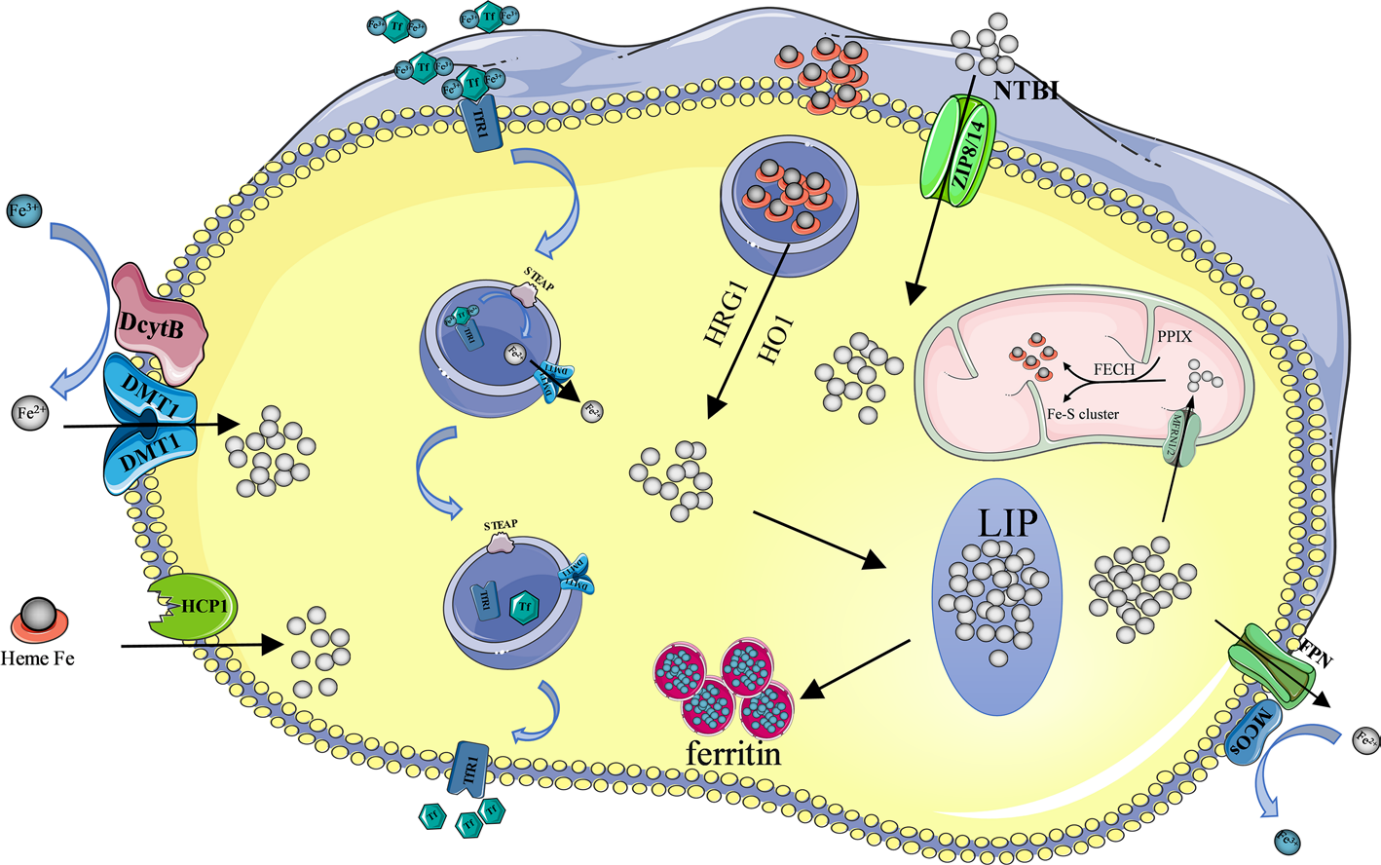
Iron metabolism in normal cells. Ferric iron in food is reduced to Fe^2+^ by DcytB and then taken up by DMT1 into the intestinal epithelium. HCP1 mediates the absorption of heme iron by intestinal cells. Fe-Tf-TfR1 complexes enter the cell by endocytosis, forming the endosome, where Fe^3+^ is reduced to Fe^2+^ by the STEAP. Then, Fe^2+^ is transported from the endosome to the cytoplasm via DMT1, and the Tf-TfR1 complex immediately returns to the cell membrane for the next cycle of transport. The figure also shows the pathway of circulating heme and NTBI into cells. *DcytB* duodenal cytochrome B, *DMT1* divalent metal transporter 1, *HCP1* Haem carrier protein 1, *LIP* Labile iron pool, *HO1* heme oxygenase 1, HRG1 heme-responsive gene 1, *FPN* ferroportin, *MCOs* multicopper oxidases, *Tf* transferrin, *TfR1* transferrin receptor 1, *STEAP* six transmembrane epithelial antigen of the prostate, *NTBI* non-transferrin-bound iron, *ZIP8/14* ZRT/IRT-like protein 8/14, *MFRN 1/2* mitoferrins ½, *PPIX* Protoporphyrin IX, *FECH* ferro chelatase

Mammalian cells obtain iron mainly through transferrin receptor 1 (TfR1). After binding to TfR1, transferrin bound iron (TBI) enters iron-requiring cells through endocytosis. Cells capture circulating heme by endocytosis and degrade heme to iron by the action of heme-responsive gene 1 (HRG1) and heme oxygenase 1 (HO1) [17, 18]. Iron can be kept free in the cell, forming an unstable iron pool (LIP). In addition, non-transferrin-bound iron (NTBI) enters cytosol LIP through multiple divalent metal transporters, including ZRT/ IRT-like protein 8 (ZIP8) or ZIP14 (Fig. 1) [19].

### Iron utilization, export and storage

Iron in the body is divided into two parts: functional state iron and storage iron. Functional status iron mainly includes: hemoglobin iron (67% of body iron), myoglobin iron (17% of body iron), transferrin iron, lactoferrin, enzyme and cofactor-bound iron. Stored iron includes ferritin and hemosiderin.

Of the iron that enters the cell, some constitutes the cytoplasmic LIP, but most enters the mitochondria via the mitoferrins (MFRN) 1 and 2 in the cell for the synthesis of heme and iron-sulfur clusters [20]. Protoporphyrin IX chelates with Fe^2+^ to form heme under the action of ferro chelatase in mitochondria. The synthesis and function of heme and iron-sulfur clusters have been extensively explored [21–23].

Intracellular iron excretion is mainly performed by ferroportin (FPN), which is expressed on the cell membrane of a variety of human tissue cells and is considered to be the only ferrous iron exporter. Iron is transported outside the cell via FPN on the basolateral membrane of the cell where it is oxidized to Fe3 + by multicopper oxidases (MCOs); then, Fe3 + binds to transferrin (Tf) to form TBI in the bloodstream [24, 25].

Unutilized and unexcreted iron in the cell is stored in the form of ferritin and hemosiderin to prevent excessive iron from damaging the cell (Fig. 1).

### Regulation of iron

The body regulates iron metabolism through hepcidin and iron-regulatory proteins (IRPs). The FPN/hepcidin system is primarily responsible for the stabilization of iron metabolism at the systemic level. Unlike TfR1, TfR2 is mainly expressed in the liver, acting as an iron sensor and regulating hepcidin production [26]. When serum iron increases, hepcidin, which is synthesized by the liver, increases and binds to FPN; this weakens the function of FPN, thus inhibiting the release of iron into the blood and reducing serum iron concentration [27–29]. The regulation of intracellular iron metabolism is mainly dependent on IRPs/iron response element (IRE) system. IRPs can be divided into IRP1 and IRP2, which have similar functions. IRPs mainly affects the expression of TfR1 and the synthesis of ferritin. When intracellular iron deficiency occurs, IRPs can bind to the iron response element on TfR1 mRNA, promoting the expression of TfR1 and inhibiting the synthesis of ferritin. IRPs also inhibits the translation of FPN, which in turn inhibits iron output. When intracellular iron overload occurs, the conformation of IRPs changes and IRPs lose their activity. Excess iron can also change the conformation of IRE and reduce the affinity of IRE to IRPs [30–32]. In addition, IRP1 and IRP2 have different functions. Overexpression of IRP1 can reduce tumour growth, while overexpression of IRP2 can have the opposite effect. It has been suggested that IRP2 has other functions that account for this different phenotype [33].

## Iron metabolism in OS

### Systemic changes in iron metabolism

As with most cancers, systemic iron metabolism is disrupted in patients with OS. OS patients often have clinical manifestations of anaemia, which is called anaemia of chronic disease, and this is caused by the inhibition of iron utilization and decreased red blood cell production [34, 35]. Interleukin-6 (IL-6) is an important factor in promoting the proliferation, metastasis and angiogenesis of OS [36, 37]. Signal transducer and activator of transcription 3 (STAT3) is overexpressed in OS cells and is related to poor OS prognosis [38, 39]. However, IL-6 and STAT3 can upregulate hepcidin and reduce serum iron concentrations [40, 41]. Tumour necrosis factor-α (TNF-α) can inhibit the synthesis of erythropoietin and plays a role in inhibiting the production of erythrocytes [42–44]. These molecules are closely related to the occurrence and development of OS and disrupts the body's normal iron metabolism by interacting with iron metabolism-related proteins, resulting in sufficient iron reserves in the body of patients with OS but reduced availability of circulating iron for red blood cell production [45, 46].

### Iron metabolism in OS cells

#### TfR1

With the discovery of more iron metabolism-related proteins and the elucidation of iron metabolism mechanisms, the relationship between iron metabolism and cancer at the molecular level has become increasingly clear [47, 48]. Due to the vigorous growth and proliferation of tumour cells, they require synthesis of a large amount of DNA in a relatively short period of time. At this point, tumour cells need much more iron than normal cells. TfR1 is an essential protein involved in iron uptake and regulation of cell growth [49]. Several studies have shown that tumour cells express higher levels of TfR1 than normal cells to increase the transport of iron to meet the metabolic requirements of tumour cells [50].

TfR1 is highly expressed in many types of malignancies, including hepatocellular carcinoma, breast cancer, leukaemia, lymphoma, lung cancer, and colorectal cancer [51–53]. Clinical trials have shown a positive correlation between elevated TfR1 expression and tumour malignancy and poorer prognosis.

**Fig. 2:**
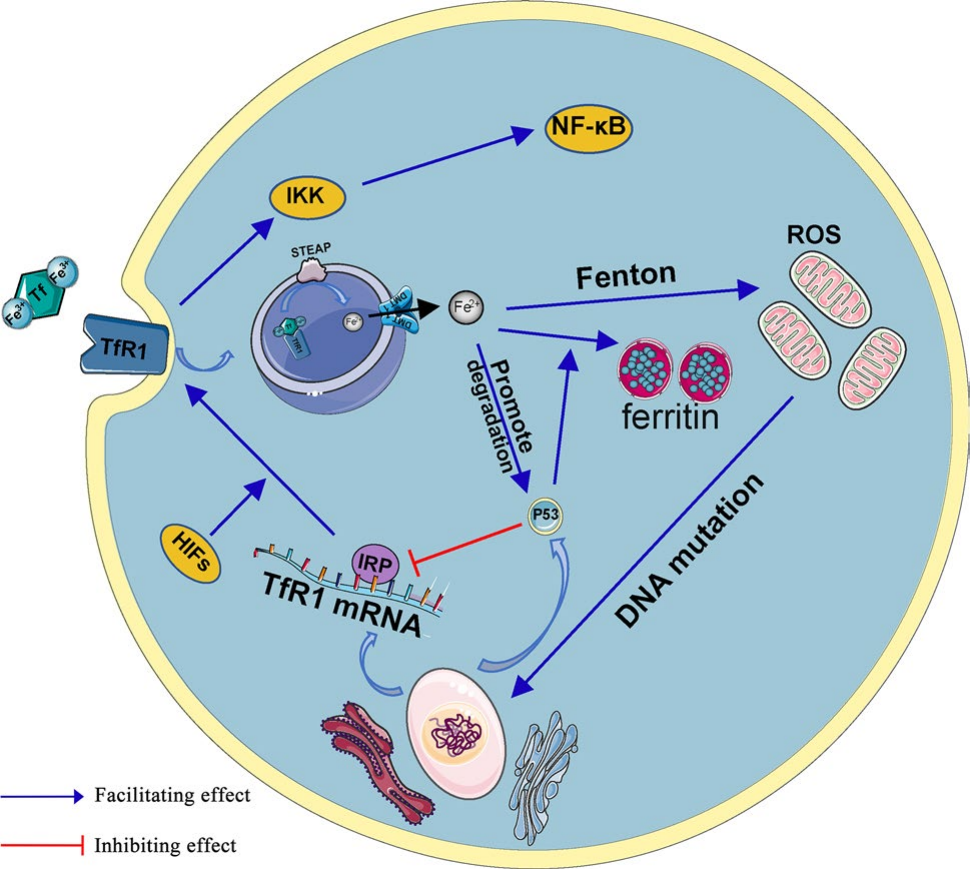
Iron metabolism in OS cells. Iron overload induces high levels of ROS production in mitochondria through the Fenton reaction, which in turn leads to DNA damage. TfR-1 interacts with the IKK complex and is involved in IKK-NF-κB signalling, thereby inhibiting OS cell apoptosis. In addition, the relationship between p53, IRP and TfR1 is also briefly depicted in the figure

Our previous study showed that TfR1 is highly expressed in OS and is significantly correlated with histological grade, Enneking stage and distant metastases; therefore, TfR1 can be used as an independent prognostic indicator for OS patients [54]. Increased expression of TfR1 increases the rate of iron uptake by OS cells, thereby promoting OS proliferation. In addition, TfR1 is involved in the regulation of the NF-kappa B (nuclear factor-kappa B) signalling pathway in cancer cells, and by interacting with IKK (inhibitor of NF-kappaB kinase), it activates the NF-kappa B signalling pathway to inhibit apoptosis, thereby promoting the survival rate of cancer cells (Fig. 2) [55].

While OS is rapidly increasing in size and lacks blood vessels in some areas of the tumour, this fraction of OS cells is chronically hypoxic, and OS cells can only adapt to hypoxia by expressing a range of hypoxia-inducible factors (HIFs). The current study showed that HIF expression is significantly increased in OS cell lines and that aberrant expression of HIF is closely associated with OS progression [56]. Activation of HIF in OS can affect cellular iron metabolism by increasing the expression levels of TfR1, inducing degradation of haem iron and promoting iron uptake to increase intracellular iron concentrations (Fig. 2) [57–59].

#### Ferritin

Ferritin is a metal-binding protein whose primary function is responsible for maintaining iron bioavailability. Ferritin exists in osteoblast cell lines and regulates metal homeostasis in bone [60]. Ferritin has two functionally distinct isoforms: ferritin light chain (FTL) and ferritin heavy chain (FTH). The level of intracellular ferritin has a certain value in the diagnosis, treatment and prognosis of osteosarcoma. A study on FTL and osteosarcoma showed that the expression of FTL in osteosarcoma cells was significantly lower than that in normal tissues in the transcriptome database. Tissue microarray analysis showed that lower FTL expression was associated with shorter tumor metastasis and survival, and the higher the FTL level, the better the treatment effect [61].

In the development of OS, TP53 is the most frequently altered gene [62, 63]. The current study found that p53 (encoded by TP53) inhibits IRPs activity, decreases TfR1 expression on the cell membrane surface, promotes intracellular ferritin synthesis, and thus restricts iron metabolism to inhibit cell growth [64, 65]. TP53 was shown to be mutated in OS cells resulting in inactivation of p53; thus, p53 could not maintain its inhibitory effect on iron metabolism, intracellular ferritin synthesis was inhibited, and intracellular free iron was increased. In an in vitro study on hepatocytes, activation of p53 was found to increase the expression of hepcidin and had an antitumor effect by reducing the intracellular concentration of iron [66]. The inactivation of p53 in OS cells may decrease the expression of hepcidin, inhibit the efflux of iron, and then increase the concentration of iron in OS cells [67]. Interestingly, high concentrations of iron can in turn contribute to the degradation of p53, creating a vicious cycle within the cell that promotes the development and progression of OS [68].

#### Free iron

Increased intracellular iron concentrations can play a role in tumorigenesis and progression by inducing high levels of ROS production in mitochondria through the Fenton reaction, causing DNA mutations [69, 70]. An in vitro study showed that iron promotes the proliferation, migration and invasion ability of OS cells and that high levels of intracellular iron increase the production of ROS in mitochondria and play a key role in the Warburg effect in OS [71]. In addition, arsenite, which has long been considered a common carcinogen, can also affect the occurrence and progression of OS by interfering with iron metabolism. Arsenite inhibits the synthesis of ferritin, which increases the concentration of free iron in OS cells. This makes OS cells more prone to ROS production, making DNA more vulnerable to damage and leading to arsenite-induced carcinogenic effects [72]. This study suggests that disturbances in iron metabolism play an important role in the progression of OS. In addition, the oxidative stress caused by iron overload depletes the body of antioxidant substances, which can also have a mutagenic effect within cells; thus, the carcinogenic effect of iron overload is cumulative and results in the transformation of normal cells to cancer cells [73].

Intracellular iron overload can also disrupt the normal functioning of the immune system, allowing tumour cells to escape immune system surveillance. In the murine fibrosarcoma cell line L929, iron overload was found to inhibit NO production in macrophages, leading to a loss of the antitumour activity of macrophages and aiding in the survival of tumour cells [74].

### Iron metabolism and treatment of OS

Given the specific role of disorders of iron metabolism in the development and progression of OS, targeting iron metabolic pathways for the treatment of OS is possible. Current attention targeting iron metabolism to treat OS has focused on ferroptosis, a non-apoptotic form of cell death caused by iron catalysis and lipid peroxidation [75]. Excessive iron not only promotes the occurrence and development of OS, but also makes OS cells, which have little response to traditional necroptosis, more susceptible to ferroptosis. Tirapazamine, curcumin and its analogues, artemisinin, etc., have shown the potential to inhibit or treat OS by inducing ferroptosis in cells [12, 76].β-phenethyl isothiocyanate (PEITC) has potential anti-cancer activity. A study involving human OS cell lines showed that PEITC affected the level of iron metabolism by up-regulating TfR1 and down-regulating iron-related proteins such as FPN, FTH1 and DMT1. In addition, PEITC also promoted ROS accumulation, activated MAPK signaling pathway, and triggered ferroptosis, thereby inhibiting or treating OS [77, 78].

In conclusion, there is great potential to treat OS by targeting iron metabolism and ferroptosis pathways.

Deferoxamine and deferasirox, two iron chelators, can alter iron metabolism in tumour tissues, activate the MAPK signalling pathway, promote ROS deposition in OS cells and induce apoptosis in OS cells, thereby reducing the viability of OS cells and inhibiting their proliferation [79, 80].

Research into the use of TfR1 for the treatment of tumour is also underway. As TfR1 is significantly differentially expressed in tumours and normal tissues, tumour cells can be identified according to their TfR1 expression levels, facilitating targeted tumour therapy. Adriamycin is the most common chemotherapy drug, but it is not selective and can cause serious damage to the heart [81]. Coupling adriamycin to TF takes advantage of TF's recognition of TfR1 to allow for precise delivery of adriamycin to cancer cells for targeted tumour therapy [82]. In addition, the TfR1 antibody can also precisely recognize TfR1, which can better identify tumour cells and block their uptake of iron due to its antitumor effects [83–85]. However, research on TfR1 in the treatment of OS is still in its infancy and faces many challenges; further in vitro and in vivo trials are needed (Table 1).

**Table 1:** Iron metabolism and treatment of OS

Compound	Target	Pharmacological Mechanisms
Tirapazamine	Suppressing SLC7A11	Inducing ferroptosis by reducing glutathione peroxidase 4 (GPX4) and increasing ROS
Curcumin and its analogues(EF24)	Increasing ROS	Inducing ferroptosis
Artemisinin	Reducing Fe2 + levels	Inducing cytotoxicity
Iron chelators	Activating the MAPK signalling pathway	Promoting ROS deposition and induce apoptosis
TfR1 antibody	Identifing and combining TfR1	Blocking iron uptake by cells
PEITC	Consuming GSH and accumulating ROS	Decreasing iron excretion, increasing iron absorption and labile iron concentration, and inducing ferroptosis
TF	Identifing and combining TfR1	Coupling drugs and targeting tumor therapy

## Conclusion

This paper briefly describes the pathways of normal iron metabolism, focusing on a review of the changes in systemic iron metabolism in OS patients, the relationship between key genes and molecules and iron metabolism-related proteins in OS cells and the application of iron metabolism in the treatment of OS. In OS, the pathways and molecules involved in iron import and storage are directly or indirectly activated, and the pathways and molecules involved in iron export are restricted and inhibited. Reverse intervention of related molecules and pathways has become a potential treatment for OS. In addition, ferroptosis mechanism is also a strategy that cannot be ignored. Excessive iron not only promotes the occurrence and development of OS, but also induces ferroptosis leading to apoptosis of OS. Therefore, how to balance the relationship between them is an urgent problem to be solved.

Due to the complexity of the molecular mechanisms of iron metabolism, there is still a relative lack of research on the mechanisms of action and signalling molecules associated with iron metabolism causing OS. In-depth studies of normal iron metabolic pathways and pathological changes in abnormal iron metabolism in OS tissues will help to further uncover the relationship between iron metabolism and the occurrence, progression, treatment and prognosis of OS, thus providing new ideas for mechanistic studies and therapeutic approaches for OS.

## Data Availability

Data sharing not applicable to this article as no datasets were generated or analysed during the current study.

## Abbreviations

OS: Osteosarcoma
ROS: Reactive oxygen species
DcytB: Duodenal cytochrome B
DMT1: Divalent metal transporter 1
HCP1: Haem carrier protein 1
TBI: Transferrin-bound iron
TfR1: Transferrin receptor 1
HO1: Heme oxygenase 1
HRG1: Heme-responsive gene 1
LIP: Labile iron pool
NTBI: Non-transferrin-bound iron
ZIP8/14: ZRT/IRT-like protein 8/14
MFRN1/2: Mitoferrins 1/2
FPN: Ferroportin
MCOs: Multicopper oxidases
Tf: Transferrin
IRPs: IRON-regulatory proteins
IRE: Iron response element
IL-6: Interleukin-6
STAT3: Signal transducer and activator of transcription 3
TNF-α: Tumor necrosis factor-α
NF-kappaB: Nuclear factor-kappaB
IKK: Inhibitor of NF-kappaB kinase
HIFs: Hypoxia-inducible factors
FTL: Ferritin light chain
FTH: Ferritin heavy chain
PEITC: β-Phenethyl isothiocyanate
